# Colonic inflammation induces changes in glucose levels through modulation of incretin system

**DOI:** 10.1007/s43440-021-00327-y

**Published:** 2021-09-17

**Authors:** Hubert Zatorski, Maciej Salaga, Marta Zielińska, Anna Mokrowiecka, Damian Jacenik, Wanda Małgorzata Krajewska, Ewa Małecka-Panas, Jakub Fichna

**Affiliations:** 1 Department of Biochemistry, Faculty of Medicine, Medical University of Lodz, Mazowiecka 6/8, 92-215 Lodz, Poland; 2 Department of Digestive Tract Diseases, Medical University of Lodz, Lodz, Poland; 3 Department of Cytobiochemistry, Faculty of Biology and Environmental Protection, University of Lodz, Lodz, Poland

**Keywords:** Crohn’s disease, DPP IV, GLP-1, Glucose metabolism, Incretins, Inflammatory bowel disease

## Abstract

**Background:**

The role of the incretin hormone, glucagon-like peptide (GLP-1), in Crohn’s disease (CD), is still poorly understood. The aim of this study was to investigate whether colitis is associated with changes in blood glucose levels and the possible involvement of the incretin system as an underlaying factor.

**Methods:**

We used a murine model of colitis induced by 2,4,6-trinitrobenzenesulfonic acid (TNBS). Macroscopic and microscopic score and expression of inflammatory cytokines were measured. The effect of colitis on glucose level was studied by measurement of fasting glucose and GLP-1, dipeptidyl peptidase IV (DPP IV) levels, prohormone convertase 1/3 (PC 1/3) and GLP-1 receptor (GLP-1R) expression in mice. We also measured the level of GLP-1, DPP IV and expression of glucagon (GCG) and PC 1/3 mRNA in serum and colon samples from healthy controls and CD patients.

**Results:**

Fasting glucose levels were increased in animals with colitis compared to controls. GLP-1 was decreased in both serum and colon of mice with colitis in comparison to the control group. DPP IV levels were significantly increased in serum, but not in the colon of mice with colitis as compared to healthy animals. Furthermore, PC 1/3 and GLP-1R expression levels were increased in mice with colitis as compared to controls. In humans, no differences were observed in fasting glucose level between healthy subjects and CD patients. GLP-1 levels were significantly decreased in the serum. Interestingly, GLP-1 level was significantly increased in colon samples of CD patients compared to healthy subjects. No significant differences in DPP IV levels in serum and colon samples were observed between groups.

**Conclusions:**

Changes in the incretin system during colitis seem to contribute to the impaired glucose levels. Differences in incretin levels seem to be modulated by degrading enzyme DPP-IV and PC 1/3. Obtained results suggest that the incretin system may become a novel therapeutic approach in the treatment of CD.

## Introduction

Crohn’s disease (CD) is a chronic, relapsing, inflammatory intestinal disorder with complex and not fully understood pathogenesis in which immunological, genetic and environmental factors play a role [1]. Nowadays, due to advances in CD therapy patients’ lifespan increased and thus, higher proportion of subjects present comorbidities such as glucose intolerance or diabetes [2]. The current gold-standard treatment of CD is based on shifting the excessive immune response and decreasing inflammation in the colon by targeting various proteins in the inflammatory cascades such as tumor necrosis factor (TNF—α), p40 subunit of interleukin 12/23 (IL 12/23) and integrin a4B7 to name only a few [3]. Furthermore, inflammatory bowel disease (IBD) therapy includes aminosalicylates, corticosteroids and immunomodulatory drugs. Nonetheless, available treatments have several limitations and side effect increasing health care costs worldwide [4, 5]. Furthermore, current IBD therapies are not sufficient and may predispose to development of comorbidities such as diabetes and cardiovascular disorders [2]. Thus, further research to understand IBD pathophysiology and search for novel therapies are required.

Enteroendocrine cells (EEC), which account for approximately 1% of all intestinal epithelial cells, constitute the largest endocrine system in human body secreting more than 30 different peptide hormones [6]. Glucagon-like peptides (GLPs), including GLP-1, are the most important bioactive compounds secreted by enteroendocrine L cells in the gut shortly after nutrient uptake [7]. GLP-1 is produced by post-translational cleavage of six amino acids from the N-terminal end of preproglucaon. The biologically active circulation forms of GLP-1 are 31-amino acid GLP-1 (7–37) and 30-amino acid GLP-1 (7–36) [8]. GLP-1 stimulates the islet B cells to secrete insulin and inhibit gastric emptying as well as reduce food ingestion [3]. Thus, it plays essential role in reducing blood glucose level and controlling body weight [7]. GLP-1 is rapidly degraded by dipeptidyl peptidase IV (DPP IV), which results in its relatively short half-live of 5–6 min in vivo [8]. Furthermore, recent research demonstrated that targeting GLP-1 signaling, by GLP-1 agonists or DPP IV inhibitors, may improve intestinal epithelial architecture and reduce inflammatory markers level in mouse models of colitis [9–15].

The primary aim of this study was to investigate whether colitis increases glucose level in mice by influencing the incretin system. Second, our goal was to assess whether colitis reduces GLP-1 level in the colon of mice and humans by inhibiting cleavage of GLP-1 precursor, pre-proglucagon or by increasing the GLP-1 degradation. We used a mouse model of colonic inflammation induced by administration of 2,4,6-dinitrobenzene-suflonic acid (TNBS). To investigate the potential involvement of the incretin system as an underlaying factor in glucose metabolism disruption, we assessed inflammatory cytokine and GLP-1 levels as well as expression of enzymes involved in GLP-1 production.

## Materials and methods

### Animals

Male C57B1/6 mice, weighting 24–28 g, were acquired from the local vivarium used for all experiments. Animals were kept under a 12-h light/dark cycle and accommodated at a constant temperature (22–23 ºC) in sawdust-lined plastic cages with free access to chow pellets and tap water. The study was performed in accordance with the guidelines presented in the Guide for the Care and Use of Laboratory Animals of the Local Ethical Committee. All experiments involving animals were accepted by the Local Ethical Committee for Animal Experiments (Protocol #64/LB03/2015). Groups of 12 mice were used in all in vivo experiments. Efforts were made to reduce the number of animals used, as well as, animal distress. ARRIVE guidelines were incorporated in all in vivo experiments (for details please see: [16]).

### Induction of colitis

Colitis was induced by intracolonic (i.c.) infusion of TNBS, as explained before [17, 18]. Mice were weighted and anesthetized with 1% isoflurane (Baxter Healthcare Corp, IL, USA). TNBS (0.1 mL of 30% ethanol in saline containing 150 mg/kg TNBS/animal) was injected through a catheter inserted 3 cm proximally from the rectum. Next, mice were held in an inclined position for 1 min to provide a through contact of the solution with the colon. Animals in control group received vehicle alone (30% ethanol in saline, replaced with equivalent volume of water instead TNBS). Previous experiments showed that the dose of TNBS used in this study is adequate to induce reproducible colitis resulting in significant macro- and microscopic damage and biochemical characteristics of colitis without causing unnecessary distress and suffering to the animals [19, 20]. Experiments were terminated by cervical dislocation 7 days after starting experiment.

### Evaluation of colonic damage

After euthanasia the colon was rapidly removed, opened longitudinally and rinsed with phosphate buffered saline (PBS) to remove the faeces. Then, immediate examination was performed as described earlier [17, 18]. Macroscopic colonic damage was determined by an established semiquantitative scoring system by adding individual scores for ulcer, shortening of the colon, colonic wall thickness, and presence of hemorrhage, faecal blood, and diarrhea. For scoring ulcer and colonic shortening the following scale was used: ulcer: 0.5 points for each 0.5 cm of ulceration. Shortening of the colon: 1 point for > 15%, 2 points for > 25% (based on a mean length of the colon in untreated mice of; *n* = 12). The colonic wall thickness was measured in millimeters and this value was added to the score. A thickness of n mm corresponded to n scoring points. The presence of hemorrhage, faecal blood, or diarrhea increase the score by 1 point for each additional feature.

### Microscopic score evaluation

Microscopic evaluation was performed as described by Krajewska et al. [21]. Segment of the distal colon (approx. 0.5 cm in length) were isolated and stapled flat, mucosal side up, onto cardboard and fixed in 10% neutral-buffered formalin for 24 h at 4 °C. After subsequent dehydration in sucrose, samples were embedded in paraffin, sectioned at 5 µm and mounted onto slides. Then, sections were stained with hematoxylin and eosin and examined using Zeiss Axio Imager setup (Zeiss, Jena, Germany) with magnification 100x. A microscopic damage score was determined in a blinded manner using the scoring system as follows: presence (score = 1) or absence (score = 0) of goblet cell depletion, presence (score = 1) or absence (score = 0) of crypt abscesses, the destruction of mucosal architecture (normal = 1, moderate = 2, extensive = 3), the extent of muscle thickening (normal = 1, moderate = 2, extensive = 3), and the presence and degree of immune cell infiltration (normal = 1, moderate = 2, transmural = 3).

### Determination of GLP-1 and DPP IV protein level by ELISA

GLP-1 and DPP IV expression in the mouse and human serum and colon was evaluated by the enzymatic immunoassay kit (competitive ELISA cat no. E-EL-H6025 (sensitivity 0.94 pg/mL) for GLP-1 and DPP IV cat no. E-EL-H0058 (sensitivity 0.19 ng/mL) for humans and GLP-1 cat no. E-EL-M3012 (sensitivity 0.10 ng/mL) and DPP IV cat no. E-EL-M2440 (sensitivity 37.50 pg/mL), Elabscience, Wuhan, China. No significant cross-reactivity in interference between mouse/human GLP-1 or DPP IV and analogues. Shortly, mouse colon samples were rinsed in ice-cold PBS to remove excess blood and feaces and weighed before the homogenization. Next, tissues were minced using motor cordless tissue grinder (Fisher scientific, Goteborg, Sweden) in the 20 volumes of ice-cold PBS. Subsequently, homogenates and serum were centrifuged for 5 min at 5000 × g, 4 °C. The supernatant and serum were used for the procedure following manufacturer’s instructions. The amount of GLP-1 and DPP IV in serum and the colonic samples was determined from the standard curve prepared with the purified GLP-1 and DPP IV standard supplied with the kit. Data of GLP-1 and DPP IV in colon samples were presented as pg per mg of colon tissue.

### Determination of TNF-α, IL-1β, IL-6, prohormone convertase 1/3 (PC 1/3) levels and GLP-1 receptor (GLP-1R) expression by Western blotting in mouse samples

Sections of the colon (10–15 mg) were isolated, washed with PBS and stored at − 80 °C until further analysis. Tissue homogenates, separation of proteins, electrotransfer of proteins, membrane incubation and bands visualization was performed as described earlier [22].

The membranes were probed with the following primary antibodies diluted in 1% non-fat dry milk in PBST for 80 min at 25 °C: mouse monoclonal anti-mouse/human TNF (1:1000; sc-52746 C-4; Santa Cruz Biotechnology, Santa Cruz, CA, USA), mouse monoclonal anti-mouse IL-6 (1:1000; sc-57315 Santa Cruz Biotechnology, Santa Cruz, CA, USA), mouse monoclonal anti-mouse/human IL-1β (1:1000, sc-515598 Santa Cruz Biotechnology, Santa Cruz, CA, USA), mouse monoclonal anti-mouse/human PC 1/3 (1:1000, WH0005122M2, Sigma Aldrich, Poznan, Poland), rabbit polyclonal anti-mouse GLP1R (1:1000, AGR-021, Alomone Labs, Jerusalem, Israel), and mouse monoclonal anti-mouse glycerylaldehyde-3-phosphate dehydrogenase (GAPDH; 1:15 000; MAB374; Merck Millipore, Warsaw, Poland) as a reference protein. Proper horseradish-peroxidase (HRP)-conjugated secondary antibody (1:6000) was applied for 1 h at room temperature. Qualitative and quantitative analysis was made by evaluating integrated optical density (IOD) by ImageLab v.5.2.1 for Windows™ program (Bio-Rad SA, Warsaw, Poland).

### mRNA expression analysis of PC 1/3 and glucagon in human samples

Total RNA was isolated using the PureLink RNA Mini kit (Life Technologies, Carlsbad, CA, USA) according to manufacturer’s protocol and described before [23]. The purity and quantity of isolated RNA were measured using dedicated spectrophotometer (BioPhotometer, Eppendorf, Germany). The RNA (1 µg) was used for cDNA synthesis using first strand cDNA synthesis kit (Fermentas, Burlington, Canada). Quantitative analysis was performed using fluorescently labelled TaqMan probes for human PC 1/3 (Hs01026107_m1), glucagon (GCG) (Hs01031536_m1) (Life Technologies, Carlsbad, CA, USA) and hypoxanthine–guanine phosphoribosyltransferase (HPRT, Hs01003267_m1), an endogenous control on Mastercycler S realplex 4 apparatus (Eppendorf, Germany). The Ct (threshold cycle) values for evaluated genes were normalized to Ct values obtained for housekeeping gene HPRT. Relative amount of mRNA copies was calculated using 2^−ΔCt^ method.

### Study population, blood and colon sample collection

The study was performed in patients of Caucasian origin (15 men and 15 women), who were admitted to Department of Digestive Tract Diseases at Medical University of (information hidden for a blinded review) from January 2016 to December 2018. Patients were allocated to two groups: CD (*n* = 15, 8 men and 7 female) and control (*n* = 15, 8 men and 7 female). Demographic characteristics of both groups are presented in Table 1. The control group included individuals subjected to colonoscopy for various indications and this was proven in histopathological report.

**Table 1:** Comparison of baseline characteristics of patients with Crohn disease and healthy subjects enrolled for the study

	Crohn’s disease	Control group	*p* value
Subjects, *n*	15 (50%)	15 (50%)	N/A
*Sex*			
Women, *n* (%)	7 (46.7%)	7 (46.7%)	1.0
Men, *n* (%)	8 (53.3%)	8 (53.3%)	
Age, years	35.67 ± 9.76	31.93 ± 8.75	0.2797
BMI, kg/m^2^	21.4 ± 0.7	21.8 ± 1.0	0.2148
Disease duration (years)	5.3 ± 3.9	N/A	N/A
*Montreal classification*			
A1/A2/A3	0/11/4	N/A	N/A
L1/L2/L3/L4	1/9/5/0		
B1/B2/B3	8/5/2		

Baseline characteristics for patients enrolled for the study. Data are presented as mean ± SEM. Comparisons between groups were performed using the Student’s *t* test (or nonparametric Mann–Whitney *U* test) and χ^2^ test

Montreal classification of Crohn’s disease: A (age at diagnosis): A1 (16 years or younger)/A2 (17–40 years)/A3 (over 40 years), L (location): L1 (terminal ileum)/ L2 (colon) /L3 (ileocolon) /L4 (upper GI), B (behaviour): B1 (non-stricturing, non-penetrating) /B2 (structuring) /B3 (penetrating)

The inclusion criteria to the study groups were based on the adequate diagnosis based on clinical, radiological, endoscopic and histopathological criteria recommended by the European Crohn’s and Colitis Organization (ECCO). Patients with CD with Crohn’s Disease Activity Index (CDAI) ≤ 220 were included into the study. CD patients receiving steroids, with history of surgery due to CD, obese patients (BMI > 25 kg/m^2^), current smokers, patients with a history of cardiovascular disease, pulmonary and kidney disease, allergy, psoriasis, atopic dermatitis were ruled out from the study.

After admission to Department of Digestive Tract Diseases about 4 mL of blood was taken from each participant issuing four vacutainer tubes. After 30 min, tubes were centrifuged for 15 min at 3000×*g* and the serum was removed. In patients, who underwent colonoscopy after admission of four colon samples from left-side colon were taken, frozen and stored until further use. The protocol was approved by Bioethical Commission (#RNN/276/15/KE).

### Statistics

Statistical analysis was performed using Prism 8.0 (GraphPad Software Inc., La Jolla, CA, USA). Data are expressed as means ± SEM. Shapiro–Wilk test was used to check the normality of data distribution. Baseline characteristics data for patients enrolled for the study are presented as mean ± SEM. Comparisons between groups were performed using the Student’s *t*test (or nonparametric Mann–Whitney *U* test) and *χ*^2^ test. *P* values < 0.05 were considered statistically significant.

**Fig. 1:**
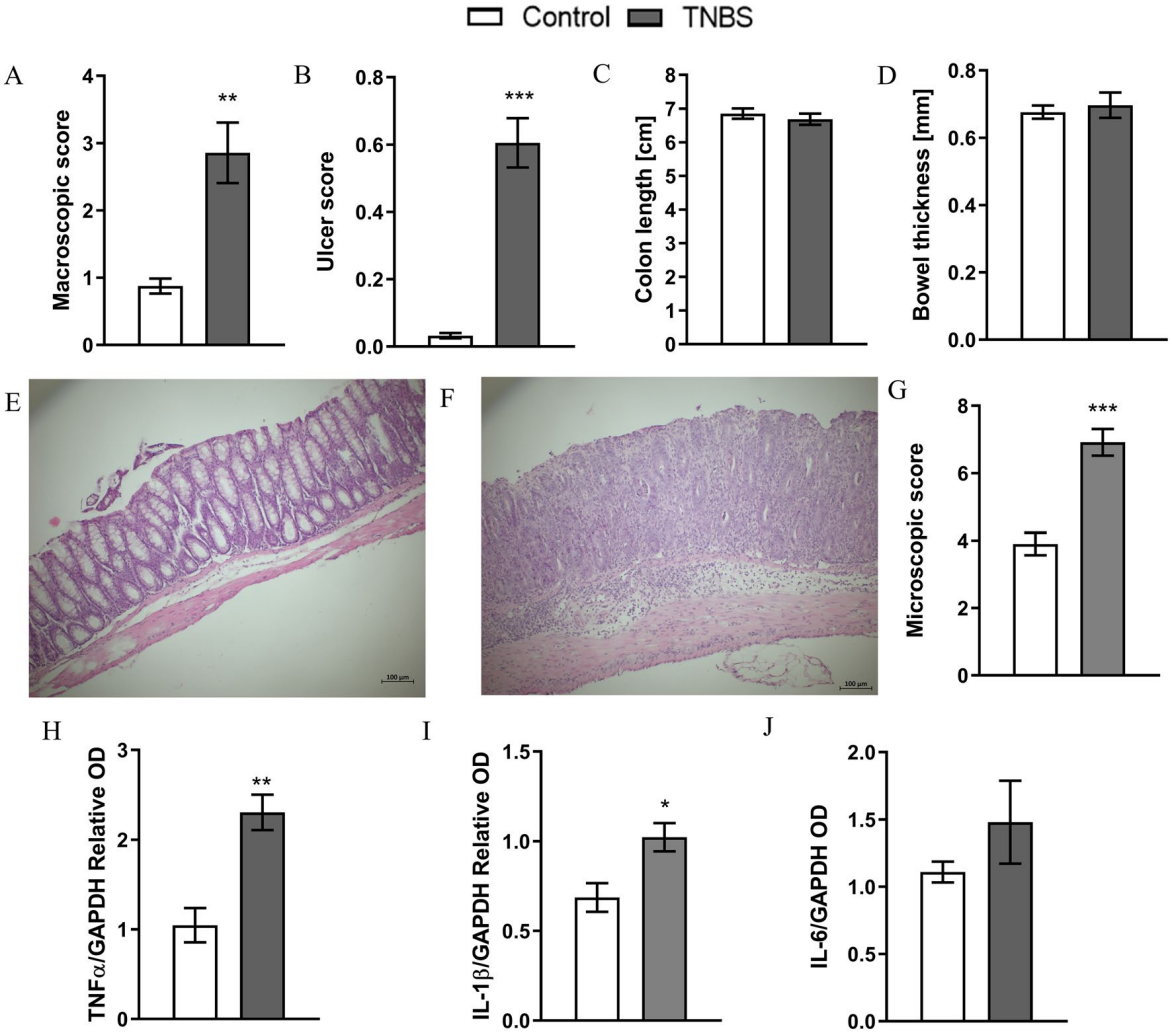
Intracolonic administration of 2,4,6-trinitrobenzenesulfonic acid (TNBS) resulted in developmeopic scoring and representative micrographs of hematont of reproducible colitis as indicated by macroscopic score (**A**) and ulcer score (**B**). No significant changes in colon length (**C**) and thickness (**D**) were observed. Microscxylin and eosin-stained sections of mouse colon from E) control and F) TNBS; **G**) Microscopic scoring. Pro-inflammatory markers in colitis. Administration of TNBS (2,4,6-trinitrobenzenesulfonic acid) significantly increased the expression of Tumor necrosis factor-α (TNF-α) (H) and Interleukin 1β (IL-1β) (**I**) and non-significantly Interleukin 6 (IL-6) (J) in the TNBS group as compared to control mice. *p < 0.05 **p < 0.01, ***p < 0.001. Results are expressed as mean ± SEM for *n* = 12 mice per group. Scale bar = 100 µm. Microscope Zeiss Axio Imager setup (Zeiss, Jena, Germany), magnification × 100. Comparison between groups were performed using Student’s *t* test. OD –optical density

**Fig. 2:**
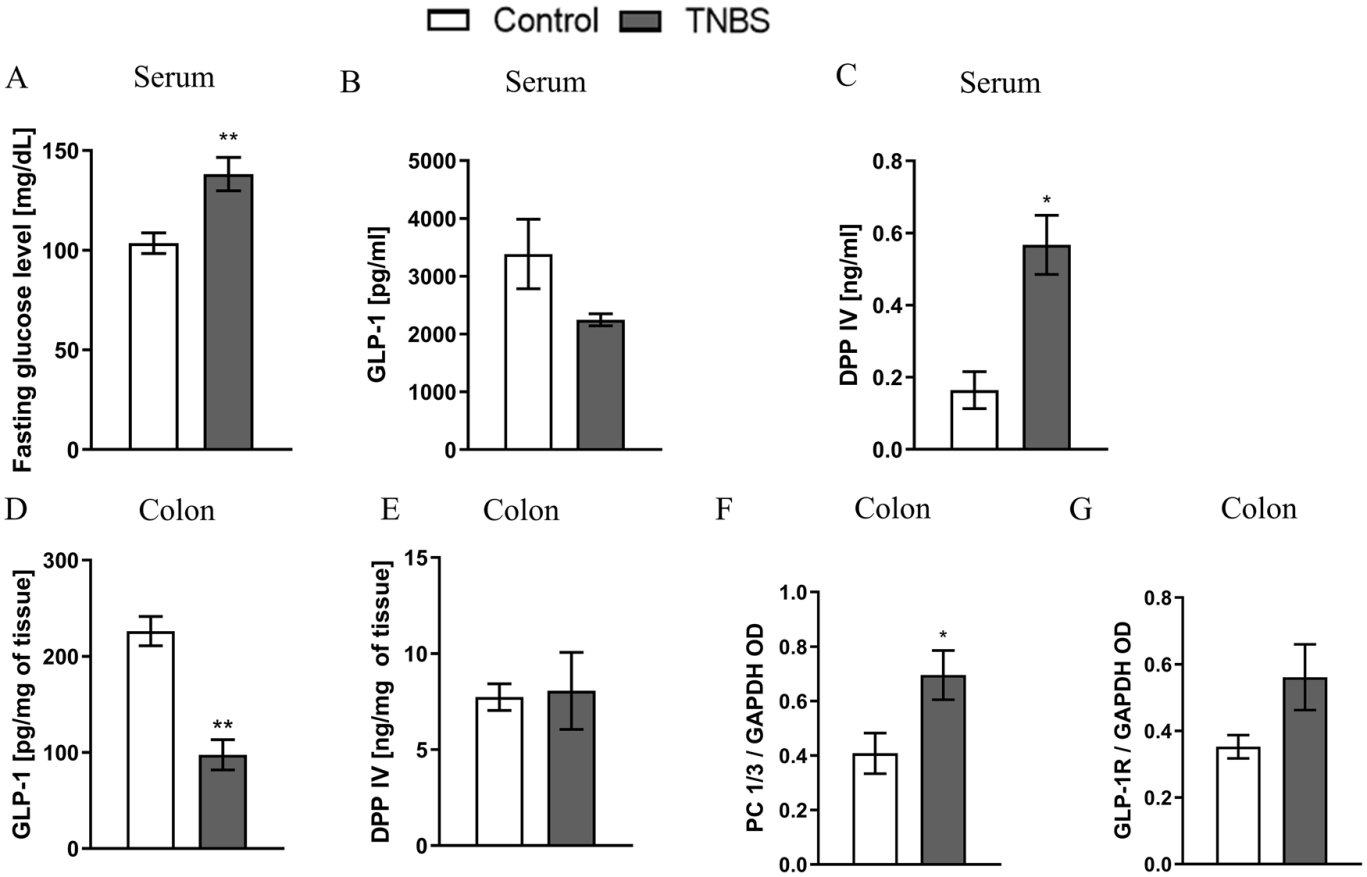
Fasting glucose level and incretin system in mice after administration of TNBS (2,4,6-trinitrobenzenesulfonic acid). Intracolonic injection of TNBS resulted in a significant increase in fasting glucose level (**A**). Glucagon-like peptide 1 (GLP-1) levels were reduced after administration of TNBS in serum (**B**) and colon samples (**D**). Dipeptidyl peptidase IV (DPP IV) levels were significantly increased in serum (**C**), but not in colon samples (**E**) in mice. Significant increase in prohormone convertase 1/3 (PC1/3) (**F**) and non-significant in Glucagon-like peptide 1 receptor (GLP-1R) (**G**) expression was observed in TNBS-treated mice in comparison to controls. *p < 0.05, **p < 0.01, ***p < 0.001. Results are expressed as mean ± SEM for n = 12 mice per group. Comparison between groups were performed using Student’s *t* test. Data of GLP-1 and DPP IV in colon samples were presented as pg or ng, respectively, per mg of colon tissue. Expression of PC1/3 and GLP-1R was calculated relative to GAPDH. OD—optical density

**Fig. 3:**
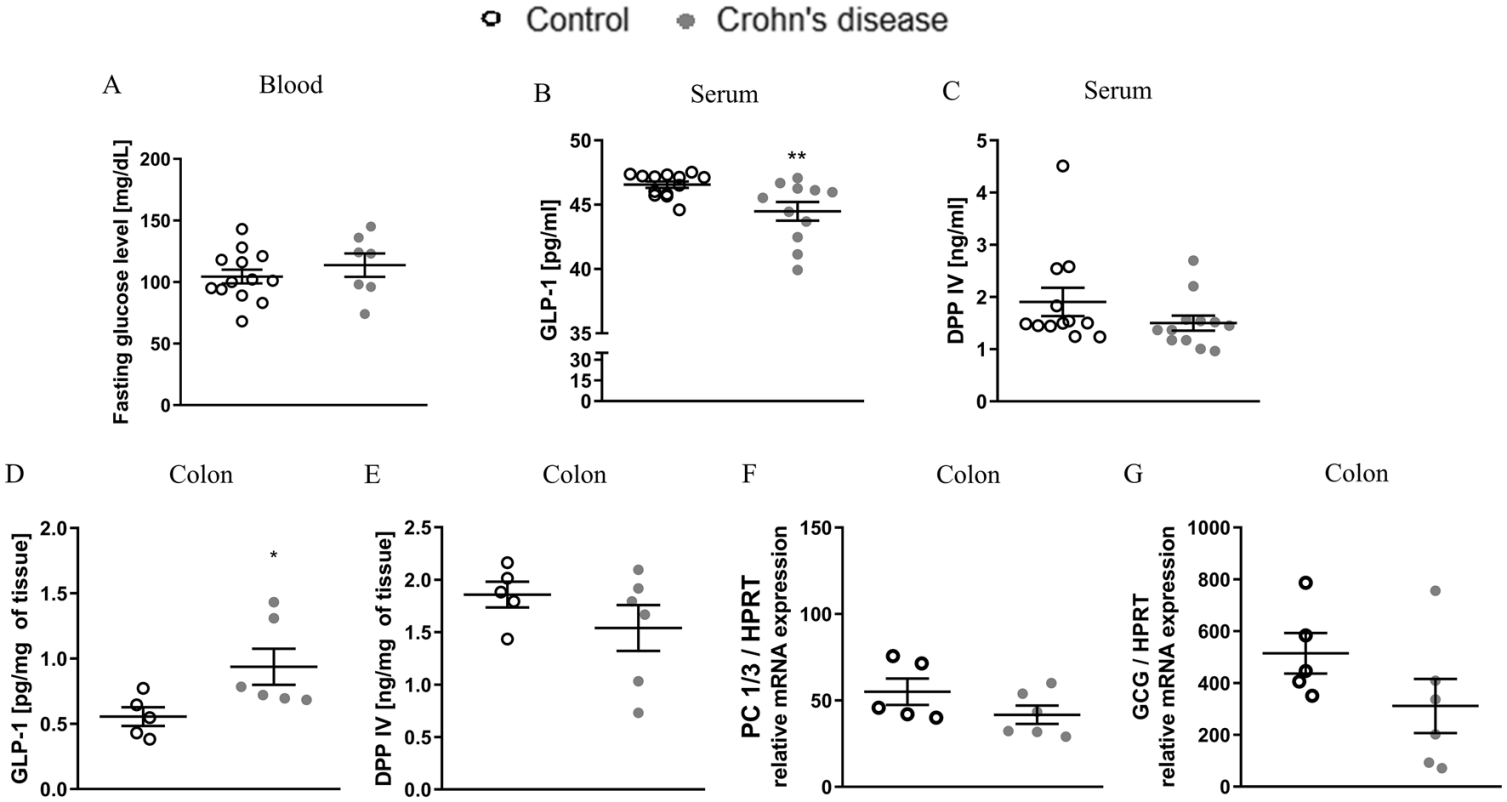
Impact of Crohn’s diseases (CD) on fasting glucose level and incretin system in human subjects. No significant difference in fasting glucose level was observed (**A**). Significantly decreased levels of glucagon-like peptide 1 (GLP-1) (**B**) and dipeptidyl peptidase IV (DPP IV) (**C**) in CD patients’ serum as compared to healthy controls. No differences in GLP-1 (**D**) and DPP IV (**E**) levels in colon samples of CD patients as compared to controls. Moreover, no differences in mRNA levels of prohormone convertase 1/3 (PC 1/3) (**F**) and glucagon (GCG) (**G**) and were observed between groups. *p < 0.05 **p < 0.01. Results are expressed as mean ± SEM for *n* = 11–15 subjects per group for serum samples and *n* = 5–6 per group for colon samples. Comparison between groups were performed using Student’s *t* test (or non-parametric Mann–Whitney *U* test). Data of GLP-1 and DPP IV in colon samples were presented as pg or ng, respectively, per mg of colon tissue. The expression of evaluated genes was normalized to expression of housekeeping gene hypoxanthine–guanine phosphoribosyltransferase (HPRT)

All data, apart from presented on Fig. 3C, D had normal distribution. Data presented in Figs. 1, 2, 3 apart from Fig. 3C, D were analyzed using Student *t* test. Data presented in Fig. 3C, D failed to have normal distribution, hence non-parametric Mann–Whitney *U* test was used.

## Results

### Induction of colitis

Administration of TNBS resulted in reproducible colitis in treated groups evidenced by significantly increased macroscopic (*t*_9_ = 3.900, *p* = 0.0036) and ulcer (*t*_7_ = 8.825 *p* < 0.0001) scores in comparison to controls (Fig. 1A, B). No differences between colon length and thickness were observed (Fig. 1C, D).

Results obtained from microscopic scoring were similar to those from gross examination of the mouse colon (*t*_9_ = 5.686, *p* = 0.0003). Administration of TNBS resulted in goblet cell depletion, destruction of mucosal architecture and increased degree of immune cell infiltration in colon samples (Fig. 1E–G).

Administration of TNBS alone resulted in a significant increase of TNF—α (*t*_5_ = 4.498, *p* = 0.0064), IL-1β (*t*_10_ = 2.899, *p* = 0.0159) and non-significant increase in IL-6 (*t*_6_ = 1.162, *p* = 0.2893) expression when compared to controls (Fig. 1H–J).

### Colonic inflammation leads to disturbances in glucose metabolism and incretin system in mice

Fasting glucose levels were significantly increased in TNBS-treated group compared to control group (Fig. 2A) (*t*_21_ = 3.439, *p* = 0.0025).

In serum, GLP-1 levels were non-significantly decreased in TNBS group as compared to control group (Fig. 2B) (*t*_7_ = 1.653, *p* = 0.1423). Moreover, serum DPP IV levels were significantly increased in TNBS as compared to controls (Fig. 2C) (*t*_5_ = 3.804, *p* = 0.0126).

In colon samples, GLP-1 levels were significantly decreased in TNBS-treated animals as compared to control group (Fig. 2D) (*t*_6_ = 5.408, *p* = 0.0017). No difference in DPP IV levels were observed between groups (Fig. 2E). Interestingly, TNBS administration resulted in a significant increase in PC 1/3 expression (Fig. 2F) (*t*_7_ = 2.367 *p* = 0.0498) and non-significant increase in GLP-1R expression (Fig. 2G) (*t*_6_ = 2.435, *p* = 0.0508) compared to control group.

### Impact of CD on blood glucose, GLP-1 and DPP IV level in humans

No significant differences were observed in fasting glucose level between healthy subjects and CD patients (Fig. 3A) (*t*_18_ = 0.8977, *p* = 0.3812). Patients with CD had significantly lower levels of GLP-1 in serum (*t*_22_ = 2.896, *p* = 0.084) in comparison to healthy subjects (Fig. 3B). Interestingly, GLP-1 level was significantly increased in colon samples of CD patients compared to healthy subjects (Fig. 3D) (*t*_9_ = 2.303, *p* = 0.0468). No significant differences in DPP IV levels in serum (Fig. 3C) and colon samples (Fig. 3E) were observed between CD individuals and control group. Moreover, there were no changes in mRNA expression of PC 1/3 (Fig. 3F) and GCG (Fig. 3G) in patients with CD, when compared to control.

## Discussion

The incidence and prevalence of IBD are still rising, currently being the highest in Western countries [24]. Nowadays, researchers pay great attention to understanding interactions between obesity, metabolic syndrome and chronic colitis [25–28]. Chronic inflammation, observed in IBD as well as excessive intake of steroids may result in improper functioning of several organs and lead to development of various comorbidities, such as glucose intolerance or diabetes similarly to observed in obese people. A study performed by Hotamisligil et al. demonstrated that the pro-inflammatory cytokine TNF—α may cause disruption in glucose metabolism by inhibiting tyrosine kinase activity in the insulin receptor leading to insulin resistance [29]. However, no research was previously conducted to investigate the role of incretin system in disturbances of glucose metabolism in IBD.

In this study, we aimed to investigate the impact of chronic inflammation on glucose levels in the mouse model of colitis. Additionally, we aimed to unravel the possible mechanism underlying disruption of glucose metabolism during colitis and to translate observations obtained in animal experiments into human conditions.

Since L type EEC are localized mainly in the ileum, we decided to use the mouse model of TNBS-induced colitis, which mimics changes in gut morphology observed in CD in humans e.g. transmural inflammation resulting in formation of ulcers, adhesion of the tissue and decreased blood flow through the gut as previously demonstrated in animal models of colitis [30–33].

We observed increased fasting glucose level in mice with colitis. Disturbances in glucose metabolism observed in our model may be triggered by chronic inflammatory state, which may cause insulin resistance. Moreover, chronic or acute state of inflammation may lead to damage of colon mucosa and EEC itself. Decreased number of EEC may be responsible for reduced total GLP-1 levels in serum and colon in mice with colitis compared to controls, which was also observed in our study. Together, insulin resistance and insufficient production of GLP-1 may be responsible for dysregulation of glucose metabolism in mice.

Noteworthy, we observed no changes in fasting glucose level in CD patients. Concurrently, GLP-1 level in these patients was significantly decreased in serum compared with control and no difference was observed in colon between CD patients and healthy subjects. Small number of patients, from whom colon samples were obtained alongside with study group characteristics (no patients with severe colitis were recruited) might have contributed to the fact that we were not able to detect all differences observed in the animal model.

GLP-1 possesses pleiotropic function acting in the endocrine and paracrine manner [7]. To date, researchers focused mainly on the anti-diabetic and anti-atherosclerotic effect of GLP-1, while its action in the GI and its anti-inflammatory effects were poorly understood. Recent research showed that patients after colectomy have increased risk of diabetes type 2 [34] and slower release of GLP-1 in response to the intake of glucose [35] indicating that there is an association between colon and GLP-1. Studies in mouse pancreatic islets showed that inflammatory cytokines, such as IL-6 induce, secretion of GLP-1 suggesting a link between inflammation and GLP-1 expression [36].

To investigate potential GLP-1 role in IBD, several studies were performed in experimental models of colitis. Yusta et al. [9] demonstrated that GLP-1R knockout mice exhibited dysregulated intestinal expression of IL-1β, IL-6 and IL-12. A study performed by Bang-Berthelsen et al. provided another proof that restoration of GLP-1 levels results in alleviation of colitis. In their study, administration of liraglutide, a GLP-1 analogue, resulted in significant improvement in histological scores and reduction of inflammatory cytokines and chemokines such as CCL20, IL-33 and IL-22 [37]. Furthermore, in another study, administration of GLP-1 coated with sterically stabilized phospholipid micelles significantly improved epithelial architecture and reduced expression of pro-inflammatory cytokines such as IL-1β in dextran sodium sulfate (DSS)-induced mice model of colitis [15].

In our study, inflammation of colon caused damage of colonic mucosa as observed in macroscopic and microscopic scoring thereby reducing the number of L cells producing GLP-1. We also observed increased expression of GLP-1R in colon samples of mice with colitis, which may be a response to decreased systemic and local levels of GLP-1. Those findings stand in line with results from another study performed by Schmidt et al. [38], who demonstrated that GLP-1 expression in colonic tissue is significantly diminished in SCID mice with adoptive transfer of CD4^+^ T cells when compared with control. On the contrary, Derosa et al. [7] showed an increased level of GLP-1, GLP-2 and glucose-dependent insulinotropic polypeptide (GIP) in rats with colitis induced by 2,4-dinitrobenzene-suflonic acid (DNBS). In addition, Kahles et al. [39] demonstrated that GLP-1 levels are significantly increased in critically ill patients admitted to intensive care unit (ICU), when compared to healthy subjects. Authors concluded that circulating GLP-1 concentrations may be increased due variety of inflammatory stimuli, including endotoxin, IL-1 and IL-6. However, while the study by Kahles et al. clearly demonstrated an association between proinflammatory markers and GLP-1 levels, the observation made in critically ill patients cannot be simply translated into IBD patients.

Primary substrates for DPP IV are GLP-1, GLP-2 and GIP (3). Apart from GLPs, DPP IV cleaves chemokines and cytokines such as IL-3, granulocyte–macrophage colony-stimulating factor (GM-CSF) [25, 40], thereby modulating the immune responses. Moreover, DPP IV exhibits non-catalytic actions through interactions with fibronectin, adenosine deaminase and caveolin-1 [25, 40]. Inhibition of DPP IV activity results in improvement in glucose tolerance. Here, we demonstrated that induction of colitis in mice resulted in a significant increase in serum DPP IV, which may be another explanation to observed differences in glucose level between controls and mice with colitis. Furthermore, our study confirmed previous data obtained by Pinto-Lopes et al. [41] who showed that DPP IV levels are decreased in plasma of IBD patients.

Importantly, inflammatory state seems to influence posttranslational processing of proglucagon. Changes in PC 1/3 expression in the mouse and human samples were observed. Moreover, no differences in proglucagon mRNA levels in CD patients in colon samples were observed in our study.

## Study limitations

This study in one from the few investigating the potential role of GLP-1 in Crohn disease pathogenesis. Nevertheless, it possesses several limitations. Authors chose the TNBS-induced model of colitis due to simplicity and reproducibility. However, TNBS administration mimics human CD only partially. Incretin hormones, such as GLP-1 are released after food intake, and about two-thirds of the insulin response to an oral glucose load is due to the potentiating effect of those hormones. Therefore, the next step could be to assess the influence of food intake on the levels of glucose, insulin and GLP-1 levels.

Furthermore, TNBS-induced model of colitis has impact on L-cells population in the colon, not reaching the ileum. Further experiments with different animal models of colitis affecting also small intestine are needed to fully investigate the role of L-cells in CD.

Moreover, induction of colitis leads to the development of inflammation and pain sensation. The sympathetic nervous system is activated, as well as the release of hormones of the CRF-ACTH-cortisol axis, glucagon, adrenaline and growth hormone. These mechanisms lead to an increase in arterial blood pressure, blood redistribution with a decrease in blood flow through the gastrointestinal tract and kidneys, and an increase in blood flow through the heart, brain and skeletal muscles. There is also an increase in the plasma level of glucose and fatty acids. The increase in plasma glucose is the result of glucose release from glycogen, gluconeogenesis, inhibition of insulin release, and a decrease in glucose consumption by cells. Unfortunately, authors could not confirm or reject the role of this mechanism in creation of fasting hyperglycemia. Further experiments are needed to assess the role of catecholamines and their metabolites, cortisol and glucagon in glucose metabolism in Crohn disease.

## Conclusions

In summary, this study demonstrated that glucose levels disruption caused by administration of TNBS in mice is correlated with GLP-1 level. The possible explanation of the observed phenomenon may involve destruction of the mucosa with L type cells and alterations in levels and activity of enzymes responsible for GLP-1 degradation such as DPP IV. Furthermore, it seems to be dependent from posttranslational processing of proglucagon by PC 1/3. Taken together with recent information about possible anti-inflammatory effect of GLP-1 we suggest that developing drugs targeting the incretin system may be potential additive therapy to IBD treatment resulting in less adverse effects and better clinical outcomes.

## Abbreviations

CD: Crohn’s disease
CDAI: Crohn’s disease activity index
DPP IV: Dipeptidyl peptidase IV
ECCO: European Crohn’s and Colitis organization
EEC: Enteroendocrine cells
GCG: Glucagon
GLP-1: Glucagon-like peptide
GLP-1R: GLP-1 receptor
GM-CSF: Granulocyte–macrophage colony-stimulating factor
HIF-α: Hypoxia-induced factor α
HPRT: Hypoxanthine–guanine phosphoribosyltransferase
i.c.: Intracolonic
IBD: Inflammatory bowel disease
ICU: Intensive care unit
NF-κB: Nuclear factor kappa B
OD: Optical density
PBS: Phosphate buffered saline
PC 1/3: Prohormone convertase 1/3
TNBS: 2,4,6-Trinitrobenzenesulfonic acid
TNF—α: Tumor necrosis factor α
VAT: Visceral adipose tissue
